# Pain in the Forecast: Investigating Weather Sensitivity Before and After Total Knee Arthroplasty

**DOI:** 10.3390/life15060847

**Published:** 2025-05-24

**Authors:** Michael Tanzer, Carl Laverdiere, Wassim Elmasry, Adam Hart

**Affiliations:** 1 Division of Orthopaedic Surgery, McGill University, Montreal, QC H3G 1A4, Canada; carl.laverdiere@mail.mcgill.ca (C.L.); adam.hart@mcgill.ca (A.H.); 2 Jo Miller Orthopaedic Research Laboratory, McGill University Health Centre, Montreal, QC H3G 1A4, Canada; 3 Faulty of Medicine, McGill University, Montreal, QC H3G 1A4, Canada; wassim.elmasry@mail.mcgill.ca

**Keywords:** weather, arthroplasty, knee, replacement, pain sensitization, weather-related pain

## Abstract

Background: While many patients report that their symptoms are influenced by weather conditions prior to their knee arthroplasty (TKA), how weather-related pain (WRP) evolves following surgery remains poorly understood. This study investigated the prevalence of WRP prior to and after TKA, assessed whether TKA resolves preoperative WRP, evaluated the incidence of new-onset WRP postoperatively, and identified associated risk factors. Methods: We prospectively surveyed 87 patients (121 TKAs) at a mean follow-up of 9 years (range: 1–26 years). Patients completed a standardized questionnaire assessing WRP before and after surgery, along with patient-reported outcome measures (WOMAC, UCLA activity score, and SF-12). Statistical analysis was performed to assess the associations between WRP and clinical or demographic variables, as well as patient-reported outcome measures (PROMs) in patients with and without WRP. Results: Preoperatively, 31% of patients (37/121 knees) reported WRP. Following TKA, WRP resolved in 48% of these cases (18/37 knees), persisted in 16% (19/121 knees), and developed de novo in 16% of patients (20 knees). Postoperative WRP was significantly associated with the presence of WRP in other joints (*p* < 0.0001), and with female sex (*p* < 0.0008). Preoperatively, patients with WRP had worse WOMAC scores for pain (*p* = 0.046), stiffness (*p* = 0.012), and physical function (*p* = 0.024). Despite these differences, all groups demonstrated significant improvement in PROMs postoperatively, with no differences between groups at final follow-up (*p* > 0.125). Conclusions: TKA leads to the resolution of WRP in nearly half of affected patients; however, a subset develops new or persistent WRP. Female sex, and multi-joint involvement are associated with WRP after TKA. These findings underscore the importance of preoperative counseling regarding expectations for pain relief, particularly in relation to weather sensitivity.

## 1. Introduction

Weather-related pain (WRP) is the association between weather changes (i.e., cold temperatures, air humidity, barometric pressure) and the occurrence of pain or abnormal sensations [1]. The connection between joint pain and weather-related conditions remains uncertain since patients are known to complain of weather-related pain, while studies addressing this relationship have reported heterogeneous results and lack consensus [2,3,4]. Although the exact mechanism by which weather can affect pain is uncertain, it is clear that both the central and peripheral thermosensitive receptors can be implicated in the weather-related pain sensitivity changes [5]. One proposed mechanism by which the weather influences pain sensitivity is through their effect on nociceptive nerve fibers. These nerve fibers are more sensitive in patients with arthritis due to elevated levels of inflammatory mediators, and can be triggered by low atmospheric pressure, joint stiffness, or subtle movements resulting from changes in temperature and pressure [6]. In addition, changes in barometric pressure have been associated with the sensitization of nerve endings [3]. Persistent nociceptive input from peripheral tissues initiates and sustains pain signaling [7]. When this input is prolonged, it can induce lasting central hyperexcitability, leading to heightened pain sensitivity and intensity. Additional hypotheses suggest that weather-related pain may result from micro-trauma to soft tissues, activation of the sympathetic nervous system, or hormonal changes involving the hypothalamic–pituitary–adrenal axis. Other contributing factors may include variations in sunlight exposure, vitamin D levels, mood, physical activity, sleep patterns, or even confirmation bias driven by the patient’s belief in a weather–pain connection [3,4,8].

In patients with knee osteoarthritis (OA), increased pain sensitivity in areas other joints than the affected knee is called systematic hyperalgesia and 20–30% of patients with knee OA have systematic hyperalgesia [5,9,10,11]. In most patients with knee OA, their preoperative widespread pain sensitivity caused by their severe pain is mostly resolved after their total knee arthroplasty (TKA) [12]. However, the study by Kim et al. on 112 TKA patients with unilateral primary OA demonstrated that TKA patients with high preoperative pain sensitivity not only had more severe preoperative pain than those with lower pain sensitivity but also exhibited more severe pain and lower PROMS 1 year postoperatively. This is relevant for TKA patients since some patients complain of persistent pain long after their TKA surgery, and since it is not unusual for a patient prior to their knee arthroplasty to indicate that their symptoms worsen with certain meteorological conditions, understanding the prevalence of WRP preoperatively and postoperatively is important to understand and deal with a patient’s residual knee pain after TKA [13,14,15,16,17,18,19,20].

Presently, there is a lack of understanding of the change, if any, in TKA patients’ WRP following their TKA. In the few limited studies that have investigated WRP in TKA patients postoperatively, they reported a prevalence of 18–56% [21,22,23,24,25,26]. However, none of these studies evaluated the ability of TKA to eliminate preoperative WRP. These studies failed to address if postoperative WRP was a result of the partial resolution of the patient’s preoperative WRP symptoms or if they occurred postoperatively, and the studies did not identify risk factors for WRP after TKA. Given that patient expectations can significantly influence the success of TKA, it is essential to assess the probability of postoperative resolution of WRP, as well as the potential risk of developing WRP following TKA [13,27,28]. The aim of this study was to prospectively determine the prevalence of WRP prior to and after TKA, assess whether TKA resolves preoperative WRP, evaluate the incidence of new-onset WRP postoperatively, and identify associated risk factors by assessing the associations between WRP and clinical or demographic variables.

## 2. Methods

The study was conducted according to the guidelines of the Declaration of Helsinki and approved by the Institutional Review Board of the McGill University Hospital Centre (MUHC) (Approval Code: 2023-9250; Approval Date: 12 December 2022). Verbal informed consent was obtained from all subjects involved in the study, as required by the MUHC Research Ethics Board.

Consecutive patients returning for their routine follow-up (at 1 year, 2 years, and every 2 years thereafter) after their primary TKA were prospectively surveyed about any WRP symptoms they were experiencing or had experienced before and after their TKA. The study exclusion criteria included revision arthroplasties, arthroplasties for oncology or fracture, and unicompartmental knee arthroplasty. All surgeries were performed by a single fellowship-trained surgeon (MT).

All patients were evaluated with a newly designed questionnaire, based on findings from the existing literature, specifically addressing any weather-related joint pain pre- or postoperatively (Appendix A). The clinical evaluation of the patient included the use of the following patient-reported outcome scores (PROMS): University of California Los Angeles (UCLA) activity level [28], Western Ontario and McMaster Universities Osteoarthritis Index (WOMAC) [29], and SF-12 in its physical (PCS) and mental (MCS) components [30].

Statistical analyses were performed using IBM SPSS Statistics (Version 29). Categorical variables were evaluated using the Chi-Square test, with a γ^2^ value below 0.05 indicating statistical significance. One-way ANOVA was used to assess associations between continuous variables and the presence of WRP. Binary logistic regression was used to calculate crude (unadjusted) odds ratios (ORs) and *p*-values for categorical comparisons. For categorical analyses, variables were dichotomized using median splits. Logistic regression models estimated odds ratios (ORs), 95% confidence intervals (CIs), and corresponding *p*-values. For both parametric and non-parametric data, effect sizes were calculated using Eta. Descriptive statistics were applied to summarize patient demographics. Paired t-tests were used to assess differences in pre- and postoperative PROMs, with a *p*-value of <0.05 considered statistically significant.

## 3. Results

A total of 121 primary TKAs (87 patients) were included in this study. The TKA patients were followed for a mean of 9 years (range, 1 to 26 years). Overall, the patients were female in 70%, involved the right knee in 52%, and the TKA was performed mainly for osteoarthritis (93%) in patients with a mean BMI of 30 kg/m^2^ (Table 1). The implants included cruciate-retaining Persona, NexGen (Zimmer, Warsaw, Indiana) and Journey (Smith & Nephew, Memphis, TN, USA). All but one of the knees were cemented. All patients received tranexamic acid perioperatively and no drains were used.

Before their TKA surgery, 31% (27/87) of the patients reported weather-related pain in one or both of their knees (37/121) (Table 2). There was complete resolution of their WRP in 48% (13/27) of the patients (18/37 TKAs) postoperatively. Sixteen percent of the patients (20 knees) who had no WRP prior to surgery developed WRP following their TKA. WRP before and after surgery was present in 16% (14/87) of the patients and knees (19/121). Of the 31 patients that had bilateral TKAs, 10 patients (20 knees) had WRP in both knees before surgery, and none of them had unilateral WRP. After surgery, five patients (50%) had their WRP completely resolved, and the other five patients (50%) had no change in their WRP. De novo WRP occurred after their surgery in 19% (6/31) of the patients with bilateral TKAs—five patients in both knees, and one patient in one knee.

ANOVA analysis found no significant associations for preoperative WRP, however, female sex (*p* < 0.0001) and other joint involvement (*p* < 0.0001) was significantly associated with postoperative WRP. Logistic analysis found that the patients with other joint involvement had 2 times higher odds of reporting preoperative WRP compared to those without other joint involvement (OR = 2.40, 95% CI: 1.07, 5.59). Other variables, including age (*p* = 0.74), weight (*p* = 0.11), female sex (*p* = 0.054), and BMI (*p* = 0.51) were not significantly associated. Postoperatively, patients with weather-related pain in other joints had 9 times higher odds of experiencing postoperative WRP (OR = 9.39, 95% CI: 3.89, 22.65, *p* < 0.0001). Additionally, female patients had 13 times greater odds of WRP (OR = 12.75, 95% CI: 2.87, 56.58, *p* = 0.008). Age (*p* = 0.70), time of follow-up (*p* = 0.66), weight (*p* = 0.52), and BMI (*p* = 0.54) were not significantly associated with postoperative WRP. Being a female was not a significant risk factor before surgery (γ^2^ = 0.084), but females were more likely to have WRP after undergoing TKA (γ^2^ < 0.001) (Table 3). BMI accounted for 98.7% (Eta = 0.987) and 97.1% (Eta = 0.971) of the differences in patients who did and did not have WRP preoperatively and postoperatively, respectively. Age accounted for 60.1% (Eta = 0.602) and 54.8% (Eta = 0.548) of the differences in whether patients experienced WRP pre- and/or postoperatively, respectively. The diagnosis leading to knee replacement, the side of surgery, and living in an urban or rural area were not associated with the occurrence of WRP.

Most commonly, patients described their WRP as intermittent in nature and occurring with rain and/or humidity (Table 4). Before surgery, 52% (14/27) of patients reporting WRP in the knee (18 knees in 14 patients) also reported WRP in more than one joint (γ^2^ = 0.062). Of the 10 patients with WRP in both their knees, 4 (40%) had WRP in other joints as well. In these patients with multiple joint WRP, the TKA failed to alleviate the WRP in 90% of the unilateral cases and in 25% of the patients with bilateral TKAs. WRP in TKA was significantly correlated with WRP in other joints (γ^2^ < 0.001). Preoperatively, the group of patients with WRP had significantly worse WOMAC pain scores, WOMAC stiffness scores, and WOMAC physical function scores (*p* = 0.046, *p* = 0.012, *p* = 0.024, respectively) than patients without WRP. Postoperatively, both the patients with and without WRP preoperatively had significant improvement in all their PROMS (*p* < 0.0001), and there was no difference between the two groups in any of the PROMS at final follow-up (*p* > 0.125) (Table 5).

## 4. Discussion

This study assessed the prevalence of weather-related pain (WRP) before and after TKA, examined whether TKA alleviates preoperative WRP, evaluated the incidence of new-onset WRP following surgery, and identified risk factors for WRP in TKA patients. The findings indicate that weather-related pain is relatively common among patients awaiting TKA, with 31% reporting WRP preoperatively. Although all patient-reported outcome measures significantly improved following surgery, WRP persisted in 52% of these patients. Notably, 16% of patients (20 knees) who did not report WRP before surgery developed it following TKA. In total, WRP was present both before and after surgery in 16% of patients (14 out of 87) and in 19 of 121 knees. Postoperative WRP was strongly associated with preoperative diagnosis, age, BMI, female sex, and the presence of WRP in other joints.

In their narrative review of weather-related joint pain following prosthetic implantation, Bongers et al. concluded that total joint arthroplasty (TJA) can be an effective intervention for mitigating weather-induced hypersensitivity [2]. However, the role in treating WRP is unclear, as there is a limited number of studies looking at the prevalence of WRP after TKA, and none of them have addressed whether or not TKA changed the patient’s symptoms. In a study of 40 patients with a minimum follow-up of 12 months following knee arthroplasty, Loth et al. found that 17.5% of the patients reported that changes in weather conditions or the season made them aware of their artificial joint [26]. At 2 year follow-up, Laskin reported that 35% of his knee arthroplasty patients had mild pain related to inclement weather [25]. In a study of 86 consecutive patients (88 knees) with osteoarthritis, mild discomfort during inclement weather was present in 21% of the patients at 5.8 years [24]. Hauer et al. specifically evaluated the frequency of WRP in a propensity score matching analysis comparing 260 TiN-coated un-cemented Advanced Coated System (ACS) TKA (Implantcast, Buxtehude, Germany) with 260 CoCr cemented Low Contact Stress System (LCS) (DePuy Synthes, Warsaw, IN, USA) TKA at 10–15 year follow-up [23]. Overall, 56% of the ACS group described their TKA as being sensitive to weather changes, compared to 21% of the LCS patients (*p* < 0.001). The authors postulated that cement limits the probability of WRP. Similarly to the previous studies, WRP was not common in our TKA patients, but more frequent than in most studies, affecting 32% of the knees at 9 year follow-up. TKA was less effective than THA in resolving preoperative WRP, with only 49% of the knees becoming asymptomatic postoperatively.

The findings in this study indicate that WRP is more common in patients prior to their TKA than in patients prior to their total hip arthroplasty (THA) [31]. Preoperatively, 31% of the TKA patients in this study reported WRP compared to 19% of the 331 THA patients in the study by Tanzer et al. THA was also found to be more effective in eliminating WRP, with a resolution rate of 71% compared to 48% in this TKA cohort. This contrasts with the retrospective chart review of 668 TKA and TKA patients by Khan et al., which found no significant association between weather variables and pain scores at 2 years postoperatively [32]. However, in that study, patients were only asked whether they were experiencing pain in their arthroplasty on the day of their postoperative visit, and this was correlated with the weather conditions on that specific day. There were no specific questions about whether the pain was weather-related or if it was present preoperatively.

Central sensitization has been proposed as a mechanism underlying weather-related pain. Persistent nociceptive input from inflamed joints can induce neuroplastic changes in the central nervous system, leading to heightened pain sensitivity and reactivity to external stimuli such as weather fluctuations [33]. Replacing the affected joint with a prosthesis may disrupt this peripheral input and improve sensitization-driven pain, as observed in 48% of the TKA cases in our study [7,12,34]. However, in some individuals, central sensitization may persist independently of peripheral triggers, resulting in a persistent, heightened state of neural reactivity [2,7,35]. Evidence from psychophysical and neuroimaging studies indicates that a subset of patients with arthritis exhibit centrally mediated pain, which correlates with poorer postoperative outcomes [21]. The degree of central sensitization and its clinical impact may vary by joint type and the extent of structural damage [22]. Prior work suggests that THA may more effectively normalize cold pain sensitivity than TKA, a finding reflected in our results, where WRP resolution was less likely in TKA than in THA [7,12,22,32]. New-onset WRP developed in 16% of knees that had no preoperative symptoms and in 58% of knee patients who had WRP in non-operative joints prior to surgery. These patterns suggest that central sensitization may not only contribute to the persistence of WRP but also to its development postoperatively. Also, the tissue damage during TKA surgery itself may act as a trigger in patients with central sensitization, promoting an exaggerated inflammatory response and increased vulnerability to meteorological changes. However, this study did not measure any neuroplastic changes or inflammatory biomarkers in the patients to confirm central sensitization.

This study has several limitations. First, patients were asked during postoperative follow-up to recall whether they had experienced WRP in their native joint prior to surgery. While all participants reported confidence in their recollections, the mean follow-up interval of nine years introduces a substantial risk of recall bias. Therefore, the findings should be interpreted with caution, particularly for patients with longer-term follow-up, as their recollection of WRP symptoms may not accurately represent their preoperative condition due to inherent limitations associated with long-term symptom recall. Second, only one patient in the cohort underwent cementless TKA, precluding any meaningful analysis of the impact of the fixation method on WRP. Overall, this study had a small cohort of patients, thereby limiting subgroup analyses, such as bilateral versus unilateral cases. Moreover, the patients were predominantly females, accounting for 70% of the cases. However, this is not different than the sex distribution in the 2023 American Joint Replacement Registry (AJRR), in which 69.8% of the patients undergoing TKA in the USA were female [36]. Additionally, patients were not evaluated for comorbid conditions such as fibromyalgia or chronic pain syndromes, which are associated with altered pain processing, central sensitization, and potentially with WRP. Lastly, the absence of a psychological assessment, despite established associations between mood disorders and preoperative WRP, represents another important limitation.

## 5. Conclusions

Weather-related pain is not uncommon among TKA patients and may persist or newly emerge postoperatively, despite overall improvements in pain and functional outcomes. Individual factors such as female sex, and multi-joint involvement appear to influence WRP outcomes. These findings emphasize the need to consider weather sensitivity in preoperative counseling and long-term pain management. As there are currently no effective treatments for WRP, future research is needed to investigate its underlying mechanisms and develop strategies to manage symptoms both before and after TKA.

## Figures and Tables

**Table 1:** Demographic information for patients undergoing TKA.

Clinical Characteristics		Total	Male	Female	Chi^2^ *
Sex, n (%)			36 (30)	85 (70)	0.084 and <0.001
Side, n (%)					0.883 and 0.509
	Left	58 (48)	20 (56)	38 (45)	
	Right	63 (52)	16 (44)	47 (55)	
Age (years)		75 ± 7	74 ± 7	75 ± 7	
Height (m)		1.65 ± 0.08	1.73 ± 0.09	1.62 ± 0.08	
Weight (lbs)		181 ± 36	195 ± 36	176 ± 36	
Body Mass Index, kg/m^2^		30 ± 5.5	29.6 ±5.5	30.5 ±5.5	
Diagnosis, n (%)					0.458 and 0.300
	Osteoarthritis	112 (93)	33 (92)	79 (93)	
	Pseudogout	2 (2)	0	2 (2)	
	Rheumatoid Arthritis	5 (4)	1 (3)	4 (5)	
	Traumatic Arthritis	2 (2)	2 (6)	0	

*: both values refer to before and after surgery, respectively.

**Table 2:** Demographic information for patients with preoperative WRP undergoing TKA.

Clinical Characteristics		Total	Male	Female
Sex, n (%)			7 (19)	30 (81)
Side, n (%)				
	Left	18 (49)	5 (28)	13 (72)
	Right	19 (51)	2 (11)	17 (89)
Age (years)		75 ± 7	71 ± 3	76 ± 6
Height (m)		1.67 ± 0.09	1.81 ± 0.07	1.63 ± 0.04
Weight (lbs)		181 ± 36	210 ± 35	178 ± 47
Body Mass Index, kg/m^2^		29.5 ± 5.5	30.1 ± 5.5	30.6 ± 5.5
Diagnosis, n (%)				
	Osteoarthritis	36 (97)	7 (100)	29 (97)
	Pseudogout	1 (3)	0	1 (3)

**Table 3:** Correlation between gender and weather-related pain before and after total knee arthroplasty.

Sex	Weather-Related Pain Before Surgery		Pearson Chi-Square (*p*-Value)	Weather-Related Pain After Surgery		Pearson Chi-Square (*p*-Value)
	Yes	No		Yes	No	
Male	7	29	11.764 (0.03)	2	34	16.695 (<0.001)
Female	30	42		37	48	

**Table 4:** Characteristics of quality of WRP for patients undergoing TKA.

	Frequency			Type of Weather Causing WRP		
WRP	All the time	Continuous	Intermittent	Cold	Rain	Humidity
Pre-surgery (37 knees)	0	4	33	12/37 (32%)	28/37 (76%)	30/37 (81%)
Post-Surgery (39 knees)	0	2	37	10/39 (26%)	21/39 (54%)	31/39 (80%)

**Table 5:** Weather-related pain and patient-reported outcome measures (PROMs).

	No WRP Preop	WRP Preop	*p*-Value	No WRP Postop	WRP Postop	*p*-Value
Clinical	52.9 ± 12	51.2 ± 10.5	0.471	79.2 ±9.3	81.7 ± 2.7	0.125
Functional	58 ± 13.5	59.9 ± 8.4	0.425	88.8 ± 15.2	92 ± 6.7	0.235
WOMAC Pain	9.7 ± 3.4	11 ± 3	0.046	0.1 ± 0.6	0.4 ± 1.7	0.149
WOMAC Stiffness	4.3 ± 1.6	5.1 ± 1.2	0.012	0.01 ± 0.1	0.1 ± 0.5	0.098
WOMAC Physical Function	32.5 ±10.2	37.3 ± 11.4	0.024	0.9 ± 3.3	1.6 ± 4.4	0.345
SF12 PCS	34.9 ± 6.1	33.1 ± 6.5	0.151	53.3 ± 2.5	53.3 ± 3.5	0.983
SF12 MCS	49.7 ± 8.5	52 ± 8.1	0.179	56.9 ± 2.8	56.5 ± 2.6	0.497
UCLA	4.2 ± 1.6	3.8 ± 1.2	0.136	5.7 ± 1	5.5 ± 1	0.490

Legend: PCS: Physical Component Summary. MCS: Mental Component Summary.

## Data Availability

No data available.

## Appendix A

Survey questions related to weather-induced joint pain:

- Did you experience weather-related hip pain prior to your knee replacement?
- Do you experience weather-related hip pain since your knee replacement?
- Rate the pain before and after on a scale of 0–10, with 0 indicating no pain; 1–3 indicating mild pain; 4–7 indicating moderate pain; and ≥8 indicating severe pain.
- How often do you experience weather-related hip pain?
- What time of day do you experience weather-related hip pain?
- What type of weather brings on the pain?
- Do you have weather pain in other joints, if so, which? Are they known to have arthritis?
- Do you live in an urban community?

## References

[1] Ferreira M.L., Zhang Y., Metcalf B., Makovey J., Bennell K.L., March L., Hunter D.J. (2016). The influence of weather on the risk of pain exacerbation in patients with knee osteoarthritis—A case-crossover study. Osteoarthr. Cartil..

[2] Bongers J., Vandenneucker H. (2020). The influence of weather conditions on osteoarthritis and joint pain after prosthetic surgery. Acta Orthop. Belg..

[3] Beukenhorst A.L., Schultz D.M., McBeth J., Sergeant J.C., Dixon W.G. (2020). Are weather conditions associated with chronic musculoskeletal pain? Review of results and methodologies. Pain.

[4] Sato J., Inagaki H., Kusui M., Yokosuka M., Ushida T. (2019). Lowering barometric pressure induces neuronal activation in the superior vestibular nucleus in mice. PLoS ONE.

[5] Fernandes G.S., Valdes A.M., Walsh D.A., Zhang W., Doherty M. (2018). Neuropathic-like knee pain and associated risk factors: A cross-sectional study in a UK community sample. Arthritis Res. Ther..

[6] Schaible H.-G., Richter F., Ebersberger A., Boettger M.K., Vanegas H., Natura G., Vazquez E., Segond von Banchet G. (2009). Joint pain. Exp. Brain Res..

[7] Kosek E., Ordeberg G. (2000). Abnormalities of somatosensory perception in patients with painful osteoarthritis normalize following successful treatment. Eur. J. Pain.

[8] Jamison R.N., Anderson K.O., Slater M.A. (1995). Weather changes and pain: Perceived influence of local climate on pain complaint in chronic pain patients. Pain.

[9] Arendt-Nielsen L., Nie H., Laursen M.B., Laursen B.S., Madeleine P., Simonsen O.H., Graven-Nielsen T. (2010). Sensitization in patients with painful knee osteoarthritis. Pain.

[10] Imamura M., Imamura S.T., Kaziyama H.H., Alves Targino R., Hsing W.T., Marques De Souza L.P., Mendonça Cutait M., Fregni F., Camanho G.L. (2008). Impact of nervous system hyperalgesia on pain, disability, and quality of life in patients with knee osteoarthritis: A controlled analysis. Arthritis Care Res. Off. J. Am. Coll. Rheumatol..

[11] Moss P., Benson H.A., Will R., Wright A. (2018). Patients with knee osteoarthritis who score highly on the PainDETECT questionnaire present with multimodality hyperalgesia, increased pain, and impaired physical function. Clin. J. Pain.

[12] Aranda-Villalobos P., Fernández-de-las-Peñas C., Navarro-Espigares J.L., Hernández-Torres E., Villalobos M., Arendt-Nielsen L., Arroyo-Morales M. (2013). Normalization of widespread pressure pain hypersensitivity after total hip replacement in patients with hip osteoarthritis is associated with clinical and functional improvements. Arthritis Rheum..

[13] Bourne R.B., Chesworth B.M., Davis A.M., Mahomed N.N., Charron K.D. (2010). Patient satisfaction after total knee arthroplasty: Who is satisfied and who is not?. Clin. Orthop. Relat. Res..

[14] Lewis G., Rice D., McNair P., Kluger M. (2015). Predictors of persistent pain after total knee arthroplasty: A systematic review and meta-analysis. Br. J. Anaesth..

[15] Koga M., Maeda A., Morioka S. (2024). Description of pain associated with persistent postoperative pain after total knee arthroplasty. Sci. Rep..

[16] Murphy J., Pak S., Shteynman L., Murphy J., Pak S., Shteynman L., Winkeler I., Jin Z., Kaczocha M., Bergese S.D. (2024). Mechanisms and preventative strategies for persistent pain following knee and hip joint replacement surgery: A narrative review. Int. J. Mol. Sci..

[17] Johns N., Naylor J., McKenzie D., Brady B., Olver J. (2024). High pain reported at 3 months post-total knee arthroplasty often persists for the next 3 years and is associated with reduced function and quality of life. Musculoskelet. Care.

[18] Caragea M., Essman M., Conger A., Quinlan N., Chalmers P., McCormick Z. (2025). Management of post-arthroplasty pain: A narrative review of emerging interventional treatments. Pain Manag..

[19] Sodhi N., Qilleri A., Aprigliano C., Danoff J.R. (2025). One Size Does Not Fit All: Women Experience More Pain Than Men after Total Knee Arthroplasty. J. Arthroplast..

[20] Sonobe T., Nikaido T., Sekiguchi M., Kaneuchi Y., Kikuchi T., Matsumoto Y. (2025). The impact of central sensitization on perioperative pain in TKA: A retrospective cohort study. Knee Surg. Relat. Res..

[21] Soni A., Wanigasekera V., Mezue M., Cooper C., Javaid M.K., Price A.J., Tracey I. (2019). Central Sensitization in Knee Osteoarthritis: Relating Presurgical Brainstem Neuroimaging and PainDETECT-Based Patient Stratification to Arthroplasty Outcome. Arthritis Rheumatol..

[22] Wylde V., Sayers A., Odutola A., Gooberman-Hill R., Dieppe P., Blom A. (2017). Central sensitization as a determinant of patients’ benefit from total hip and knee replacement. Eur. J. Pain.

[23] Hauer G., Leitner L., Ackerl M.C., Klim S., Vielgut I., Ehall R., Glehr M., Leithner A., Sadoghi P. (2020). Titanium-nitride coating does not result in a better clinical outcome compared to conventional cobalt-chromium total knee arthroplasty after a long-term follow-up: A propensity score matching analysis. Coatings.

[24] Laskin R.S. (2008). The Classic: Modular Total Knee-Replacement Arthroplasty. A Review of Eighty-nine Patients. Clin. Orthop. Relat. Res..

[25] Laskin R.S., Davis J. (2005). Total knee replacement using the Genesis II prosthesis: A 5-year follow up study of the first 100 consecutive cases. Knee.

[26] Loth F., Liebensteiner M., Giesinger J., Giesinger K., Bliem H., Holzner B. (2018). What makes patients aware of their artificial knee joint?. BMC Musculoskelet. Disord..

[27] Mancuso C.A., Sculco T.P., Wickiewicz T.L., Jones E.C., Robbins L., Warren R.F., Williams-Russo P. (2001). Patients’ expectations of knee surgery. J. Bone Joint Surg. Am..

[28] Amstutz H.C., Thomas B.J., Jinnah R., Kim W., Grogan T., Yale C. (1984). Treatment of primary osteoarthritis of the hip. A comparison of total joint and surface replacement arthroplasty. J. Bone Joint Surg. Am..

[29] Bellamy N., Buchanan W.W., Goldsmith C.H., Campbell J., Stitt L.W. (1988). Validation study of WOMAC: A health status instrument for measuring clinically important patient relevant outcomes to antirheumatic drug therapy in patients with osteoarthritis of the hip or knee. J. Rheumatol..

[30] Ware J.J., Kosinski M., Keller S.D. (1996). A 12-Item Short-Form Health Survey: Construction of scales and preliminary tests of reliability and validity. Med. Care.

[31] Tanzer M., Laverdiere C., Elmasry W., Hart A. (2025). Weathering the Storm: The Impact of Total Hip Arthroplasty on Weather-Related Pain. Bone Joint J..

[32] Khan S., Pescatore S.M., Panchbhavi V.K. (2025). Does Weather Have an Influence on Pain in Patients with Arthroplasty?. Musculoskeletal Care.

[33] Schaible H.-G. (2025). Afferente Matrix und periphere Sensitivierung. Man. Med..

[34] Graven-Nielsen T., Wodehouse T., Langford R.M., Arendt-Nielsen L., Kidd B.L. (2012). Normalization of widespread hyperesthesia and facilitated spatial summation of deep-tissue pain in knee osteoarthritis patients after knee replacement. Arthritis Rheum..

[35] Volcheck M.M., Graham S.M., Fleming K.C., Mohabbat A.B., Luedtke C.A. (2023). Central sensitization, chronic pain, and other symptoms: Better understanding, better management. Clevel. Clin. J. Med..

[36] Ryan S.P., Stambough J.B., Huddleston J.I., Levine B.R. (2024). Highlights of the 2023 American Joint Replacement Registry Annual Report. Arthroplast. Today.

